# Urbanisation modulates plant-pollinator interactions in invasive vs. native plant species

**DOI:** 10.1038/s41598-019-42884-6

**Published:** 2019-04-23

**Authors:** Sascha Buchholz, Ingo Kowarik

**Affiliations:** 1 Department of Ecology, Technische Universität Berlin, 12165 Berlin, Germany; 2 Berlin-Brandenburg Institute of Advanced Biodiversity Research (BBIB), 14195 Berlin, Germany

**Keywords:** Invasive species, Urban ecology

## Abstract

Pollination is a key ecological process, and invasive alien plant species have been shown to significantly affect plant-pollinator interactions. Yet, the role of the environmental context in modulating such processes is understudied. As urbanisation is a major component of global change, being associated with a range of stressors (e.g. heat, pollution, habitat isolation), we tested whether the attractiveness of a common invasive alien plant (*Robinia pseudoacacia*, black locust) vs. a common native plant (*Cytisus scoparius*, common broom) for pollinators changes with increasing urbanisation. We exposed blossoms of both species along an urbanisation gradient and quantified different types of pollinator interaction with the flowers. Both species attracted a broad range of pollinators, with significantly more visits for *R. pseudoacacia*, but without significant differences in numbers of insects that immediately accessed the flowers. However, compared to native *Cytisus*, more pollinators only hovered in front of flowers of invasive *Robinia* without visiting those subsequently. The decision rate to enter flowers of the invasive species decreased with increasing urbanisation. This suggests that while invasive *Robinia* still attracts many pollinators in urban settings attractiveness may decrease with increasing urban stressors. Results indicated future directions to deconstruct the role of different stressors in modulating plant-pollinator interactions, and they have implications for urban development since *Robinia* can be still considered as a “pollinator-friendly” tree for certain urban settings.

## Introduction

Invasive alien plant species have been reported to be a major driver of change in altering biodiversity patterns^1–3^. By contrast, a recent meta-analysis reveals largely reducing or neutral effects of invasive plants on animal abundance, diversity, fitness, and ecosystem processes^4^. Responses of native insects to invasive plants are mostly ambiguous^5^, and include negative^6,7^ as well as positive effects^8,9^. By modulating plant-pollinator networks^10–12^, invasive plants can also affect key ecological processes with a high relevance for plant reproduction and thus for agriculture, food production and food security^13–15^, even in urban environments^16,17^.

**Figure 1:**
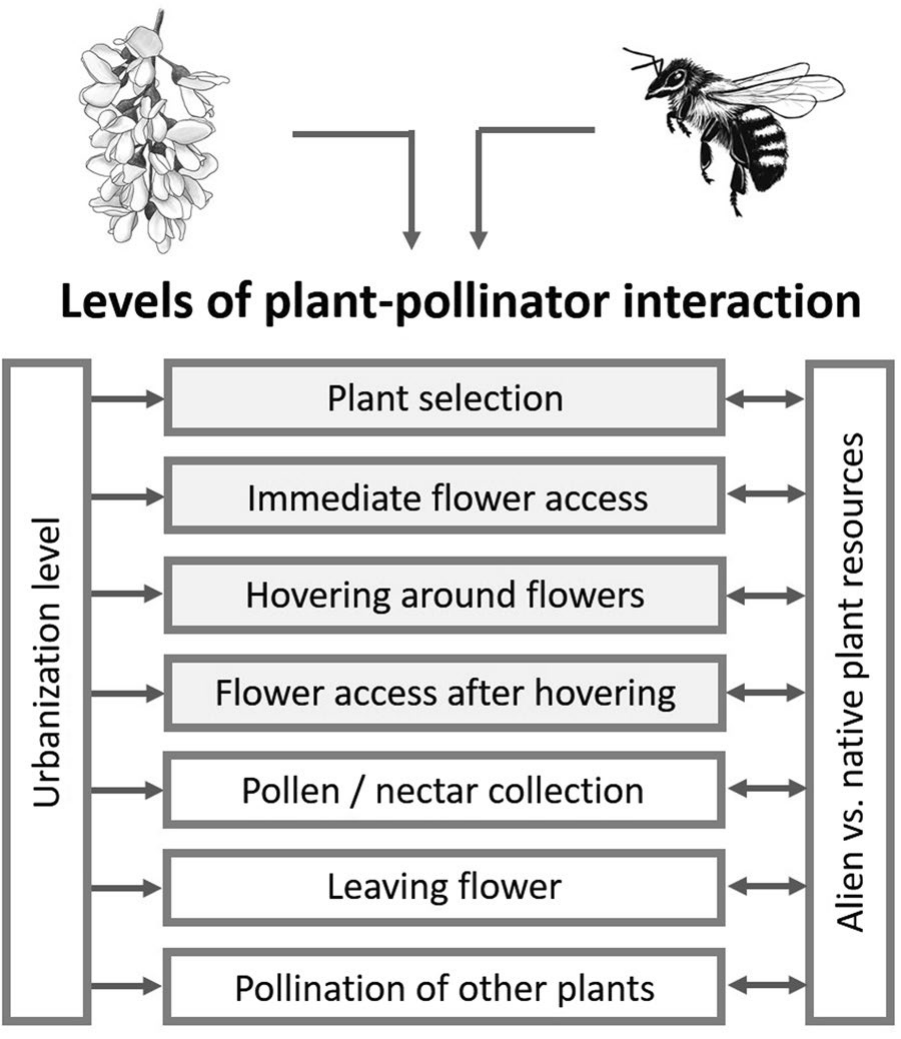
Generalised sequence of plant-pollinator interaction in relation to decisions between using native vs. alien plant resources and potential interactions with different levels of urbanisation. Levels in filled boxes were addressed in this study.

Plant-pollinator interactions comprise several interlinked processes that result in different levels (Fig. 1)^18,19^. The first is, from the pollinator perspective, the selection of a plant as a floral resource, followed by access to the flower, either immediately or after a period of hovering around the florescence (Fig. 1). After accessing the flower, pollen or nectar can then be collected and carried to its nest or another plant.

Previous studies showed that alien plant species can significantly modulate important components within plant-pollinator interactions and networks^20–22^. For example, plant selection and blossom access can differ between invasive and native plants^19,23–25^, with multidirectional patterns as pollinators preferring either native or invasive plants^18^, or in some cases neither^26^. Studies on pollen and nectar collection found that food resources of invasive plants can be either neglected by native pollinators^27,28^ or accepted as new foraging alternatives^29,30^. Due to competition for pollinators, the presence of invasive plants can affect flower visits^10,31^ and the pollination success of native plants, again with different outcomes^22,32^.

Given the multidirectional effects of invasive plants on plant-pollinator networks^18,22,33^, understanding the underlying mechanisms is a key challenge. Previous studies revealed a range of mechanisms related, for example, to flower morphology^34^, nectar chemistry and pollen quality^35^, spatial scale^36^, and biological plasticity of pollinating species^9^.

However, effects of different environmental settings on pollinator interactions have received less attention^37^. This is an important research question as recent studies increasingly evidence the role of stressors related to climate change^38,39^, land-use changes^40,41^ or urbanisation^42–44^ in modulating plant-pollinator interactions and networks^45^. Whether varying levels of urbanisation and associated stressors (e.g. heat, pollution, habitat isolation) affect the attractiveness of native vs. alien plants for pollinators could be revealed by applying a standardised study design with pairwise alien/native comparisons. To the best of our knowledge, such studies are missing thus far. This is a vital knowledge gap as cities are hotspots of alien plant species^46,47^. At the same time, cities have increased in importance as habitats for pollinators^48,49^ with a conspicuous decline in rural settings^50,51^. A better understanding of interactions between invasive plants, urbanisation and pollinators will shed light on understudied mechanisms in plant-pollinator networks^22^ and could support pollinator-friendly urban conservation policies^52^.

Therefore, we tested whether urbanisation modulated the attractiveness of an invasive vs. a native plant species for pollinators at different interaction levels, as indicated in Fig. 1. In a standardised pair-wise approach, we exposed blossoms of *Robinia pseudoacacia* L. (black locust; henceforth *Robinia*) and *Cytisus scoparius* (L.) (common broom*;* henceforth *Cytisus*) to the same type of ecosystem along an urbanisation gradient in Berlin. While the first species is native to North America and has been classified as invasive in Europe^53^, the latter is native to Europe, but invasive elsewhere^54^. Both species share biological features that are relevant for pollinators, such as flower morphology and attractive flower colour^34^. We quantified plant choice and accessing of flowers by native pollinators through direct observation. We differentiated between: (i) immediate blossom access, (ii) hovering around flowers without blossom access, and (iii) blossom access after hovering (Fig. 1). Environmental conditions regarding plant community, flower coverage and maintenance were similar at each study site, except that the location was different in relation to levels of urbanisation (Appendix 1).

We hypothesised that the native and alien plants did not differ in their attractiveness for pollinators since they shared the same flower morphology, flowering time, and both had highly attractive flower colours. We therefore expected no significant differences in plant contact of any type, immediate blossom access, only hovering around flowers, or blossom access after hovering. Since urbanisation and related environmental stressors might affect plant-pollinator interactions, we also hypothesised that the attractiveness of the invasive plant for pollinators will decrease with increasing urbanisation. Extent of green area within the city positively affects plant-pollinator interactions in terms of bee visitation which implies adverse effects due to an increased amount of impervious area^43^. Mutualisms that have evolved over long evolutionary time scales – as in the case of native plants and native pollinators – might be more resilient to anthropogenic disturbances or stressors than younger ones^55–57^. We therefore expect that younger mutualisms – for example between native pollinators and alien plants – might have a lower resilience and a more prone to disturbance which in turn should result in reduced interactions such as visitation rates. Consequently, we expected pollinators to select *Robinia* less frequently with urban than non-urban sites.

## Results

### General results

A broad range of pollinator taxa visited flowers of both the native and the alien plant species. Pollinators included, with decreasing abundance Diptera s. l. (147), Hymenoptera s. l. (114), bumblebees (24), honey bees (17), and beetles (12), while hoverflies, wild bees, butterflies and wasps played a minor role (Appendix 2).

### Attractiveness of *Robinia* and *Cytisus* for pollinators

**Figure 2:**
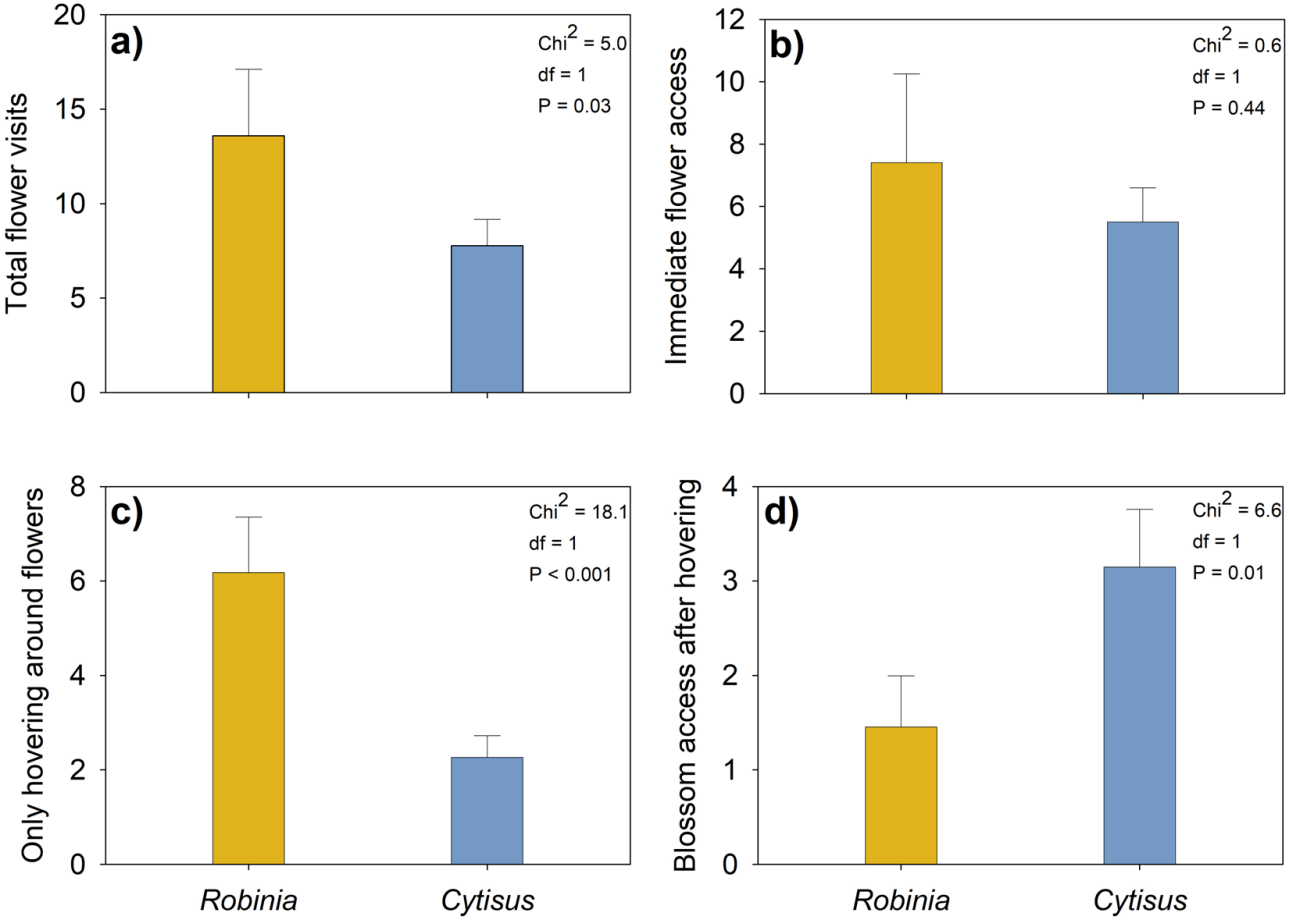
Total flower visits (=summation of the following categories b, c, and d) differed significantly between the invasive *Robinia pseudoacacia* and the native *Cytisus scoparia* **(a)**; while immediate flower access was not significantly different **(b)**. Hovering around flowers was more frequent in the presence of *R. pseudoacacia* **(c)**; but pollinators decided upon contact with *C. scoparia* more often after hovering **(d**; GLMM with Gaussian distribution).

*Robinia* attracted significantly more pollinators than *Cytisus* (Chi^2^ = 5.0, df = 1, P = 0.03; generalised linear mixed model, GLMM; Fig. 2a). However, the number of immediate flower access attempts was the same for both species (Chi^2^ = 0.6, df = 1, P = 0.44; GLMM; Fig. 2b). In contrast, the number of pollinators that only hovered around a flower without directly contacting it was significantly higher for *Robinia* than for *Cytisus* (Chi^2^ = 18.1, df = 1, P < 0.001; GLMM; Fig. 2c). The decision rate for contact with flowers after hovering was significantly higher for *Cytisus* compared to *Robinia* (Chi^2^ = 6.6, df = 1, P = 0.01; GLMM; Fig. 2d).

### Urbanisation and attractiveness of *Robinia*

**Figure 3:**
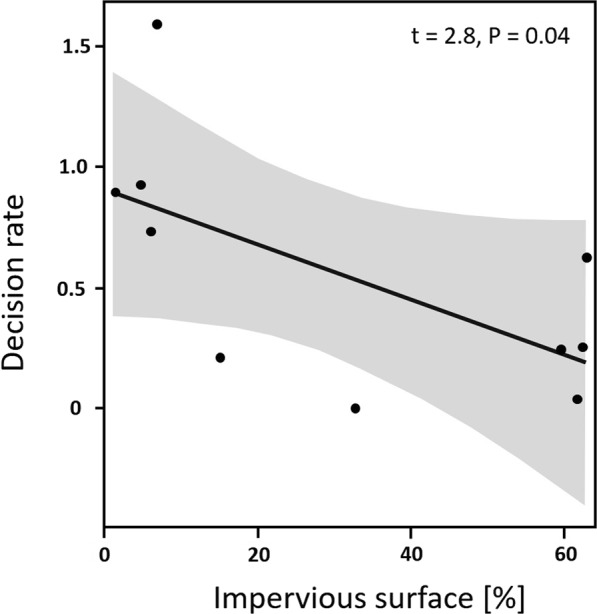
Decision to visit flowers of *Robinia pseudoacacia* after hovering in front of the florescence significantly decreased with increasing levels of urbanisation (GLMM with Gaussian distribution).

Urbanisation was not correlated with total flower visits, immediate blossom access and hovering around flowers. Yet, we found a significant urbanisation effect on plant-pollinator interactions as the decision to visit flowers of *Robinia* after hovering decreased with increasing percentage of impervious area around the study site (t = 2.8, P = 0.04; generalised linear model, GLM; Fig. 3). No other environmental variable affected any of the response variables.

## Discussion

Cities are important habitats for pollinators^49,58^, and previous research has revealed urbanisation effects on the composition of wild bee populations^59–63^. Moreover, it has been suggested that the high presence of alien plant species in cities^46,47^ negatively affects native pollinators due to problems of accessibility with novel flower types or because of differences in the quality of nectar or pollen^18,35^. However, to what extent urbanisation modulates interactions of native pollinators with alien vs. native plants remains a critical knowledge gap^37,64^. Pollinator observations in urban environments have yielded important insights into plant-pollinator interactions^29,43,44,65–68^, although these studies did not directly compare native vs. invasive species. This study took a step forward by analysing the interacting effects of biological invasion and urbanisation on plant-pollinator interactions.

### Attractiveness of *Robinia* to native pollinators

Alien plant species have been demonstrated to provide valuable floral resources for pollinators in urban environments^30,33^, including *Robinia* in Berlin^44^ and Paris^29^ and *Cytisus* in its non-native North American range^69^. A previous study found weak negative effects of *R. pseudoacacia* on urban insect communities^70^. Yet, *Robinia* strongly invests in reproductive organs, producing a large flower crop and valuable nectar resource^29,71^. However, the capacity of an invasive species to provide suitable resources can be different in native and novel landscapes^72^. While honeybees had been reported as major pollinators^53^, a recent study reveals a range of wild bee species also visits flowers of urban *Robinia* trees^44^. Our study documented that floral resources of *Robinia* attracted an even broader range of pollinator taxa in urban settings, including wild bees and flies, as well as honey bees (Appendix 2). In addition to the attractiveness of its floral resources, the long presence of *Robinia* in Europe over a period of >350 years^53^ might have helped to integrate the alien plant into native pollinator communities, as has been shown in other cases^5,73^.

Due to its large flower crop, *Robinia* provided abundant nectar resources which attracted different native pollinators among the Diptera and Hymenoptera. The number of total flower visits was significantly higher for *Robinia* compared to *Cytisus* (Fig. 2a), while there was not a significant difference in immediate flower access (Fig. 2b). Acceptance of invasive plants as food resource in the presence of a native alternative has been documented previously for urban habitats^29,33,44^. However, as a surprising result of our study, more pollinators hovered significantly longer in front of *Robinia* flowers (Fig. 2c), and significantly less decided to visit the alien flowers after hovering compared to those that hovered in front of native *Cytisus* flowers (Fig. 2d). Both species were presented in an array that ensured free plant choice and easy access. Given that *Robinia* and *Cytisus* shared a similar floral morphology and blossom colours, that are very attractive and easily recognized by pollinators, these results might indicate a trade-off behaviour. This means that pollinators have to make economic choices about what type of flowers they visit to increase benefits (energy intake) compared to costs (energy consumption)^74^. Due to these energetic requirements nutrient availability in nectar or pollen plays a vital role^30,35^. Therefore, one reason for lower decision rates for *Robinia* might be related to quality and suitability of the nectar which might be superior in *Cytisus* compared to *Robinia*. Although honey bees successfully process nectar of *Robinia*^75^ other non-domesticated and more specialised pollinators could be more sensitive in their nutritional requirements^30^. In that case, longer hovering in front of *Robinia* flowers without subsequent flower visits could hint at an ecological trap^76^, since this behaviour could disrupt the metabolic cost/benefit balance, energy expended for no reward^77^. However, analysis of *Robinia* nectar and pollen quality did not support this hypothesis, revealing high contents of suitable amino acids, phytosterol and sugar^29^. Apart from energetic requirements that affect flower selection, pollinators are faced with other economic choices when choosing a species or not such as risk-sensitivity to predators, mate searching, nest provisioning, distance to nest, floral landscape features and flower handling^74^. For example, despite obvious similar floral morphology foraging for nectar and pollen on alien *Robinia* might result in lower load sizes or longer handling times making that species less attractive in harsher environments due to higher costs.

### Urbanisation and plant-pollinator interaction

It is well established that urbanisation modulates biodiversity patterns across a range of taxa^78,79^, including pollinators^61,80–82^. Our results suggest that biological invasion and urbanisation might jointly affect plant-pollinator interactions. While *Robinia* did attract a broad range of pollinators in urban settings, similar to an attractive native plant (Appendix 2), decisions of pollinators to visit *Robinia* flowers after hovering in front of the florescence decreased significantly with increasing levels of urbanisation (Fig. 3), unlike for the native *Cytisus* (results not shown). This was consistent with Hausmann *et al*.^44^, who generally found fewer flower visits at trees (including *Robinia*) in urban settings; however, they did not differentiate between alien and native species in pairwise comparisons.

Why might increasing urbanisation make a generally highly attractive alien plant species less attractive to pollinators? Urbanisation related stressors, such as heat, pollution and habitat fragmentation^38^, might combine to produce harsher environmental conditions for pollinators that could translate into changes in biotic interactions^83,84^, including feeding behaviour and food choice along rural-urban gradients^85^. For example, urbanisation and related higher amount of impervious area might generally reduce bee visitation rates in cities^43^. Our study detected some negative effects of urbanization in flower visitation in the invasive plant species, but not in its native counterpart. This might be explained by a shorter time of co-evolution between alien vs. native plants and native pollinators making interactions less resilient against environmental stress - in line with theories of Sachs & Simms^55^ and Kiers *et al*.^56^ on mutualisms in a changing world. These authors assume that mutualisms that have evolved over long time scales are more resilient to anthropogenic impacts compared to more recently established mutualisms. At first appearance, *Robinia* might be attractive by offering large flower crops which resulted in higher numbers of hovering. Yet a shorter evolutionary experience in using food resources of the alien vs. the native species might translate to lower decision rates to visit the alien plant.

As the community structure of wild bees can change with urbanisation^59–62^, different pollinators with different food preferences might be present in rural vs. urban settings. Such changes might affect plant-pollinator interactions. However, we assume a low effect of species turnover, since the percentage of more common and less specialised species usually increases with urbanisation^59,68,86^. In our study, environmental constraints (i.e. heavy rainfalls) reduced the flowering time of *Robinia* which led to a relatively low number of replications. We addressed that by maximising the observation periods per site to increase the validity of our data.

Our study has implications for future initiatives. While we found a significant relationship between urbanisation and changes in interaction between a native pollinator and a common alien invasive plant, the roles of the different urban stressors were not determined. Unlike many native tree species that might not grow successfully in harsh urban environments^87^, *Robinia* is well adapted to such conditions and a warmer climate^88^. Despite a decreasing attractiveness of *Robinia* along with increasing urbanisation, this alien tree species still remained attractive for many pollinators. *Robinia* can thus be considered as a “pollinator-friendly” tree for urban settings – particularly when native alternatives are excluded by harsh urban environments.

## Material and Methods

### Study area and study system

The study was performed in Berlin, Germany, which has an area of 892 km² and about 3.6 million inhabitants. The climate was temperate, with an annual mean temperature of 9.9 °C and a mean precipitation of 576 mm (reference period: 1981–2010)^89^. Berlin represented a complex urban matrix with a variety of land uses, consisting of roughly 54% built-up areas, 21% woodland, 12% green space, 6% water, 5% grassland and 2% arable fields^90^. We used urban grasslands as the study system, as these ecosystems are known as important habitats for wild bees^49,61,91^ and represent a major component of Berlin’s greenspace system^92^.

To test for effects of urbanisation on the attractiveness of invasive vs. native species for pollinators, we chose *Robinia* as a model of an invasive plant as it has abundantly colonized a range of ecosystem types in central Europe, including cities^53,93^. In Berlin, *Robinia* was present in a range of habitats across the city, also forming extensive stands^78,94^.

As different flower symmetry could mask other effects, such as different flower accessibility, and therefore hamper pair-wise comparisons^34,95^, we chose *Cytisus* as a native reference species for the comparative plot-design. *Cytisus* and *Robinia* were both members of the Fabaceae, and shared similar flower morphology, blooming period, and flower colour (i.e., yellow and white), that usually attracted many pollinators (see Galloni *et al*.^96^ for *Cytisus*). Both species have been present in Berlin over a period of >300 years and have colonized a range of ecosystem types^94^, sometimes co-habiting vacant land and transition zones between pioneer forests and dry grassland.

### Study design

The study was conducted at 10 sites (Appendix 1) that were all located in urban dry grassland, within a minimal distance of 1 km to avoid nestedness. Ten study sites have been demonstrated to be a meaningful sample size for direct pollinator observations^44^. To assess the effect of urbanisation on pollinator-plant contacts, grassland sites were assigned to different levels of urbanisation. This was determined by the amount of impervious surface, the human population density and the density of roads in a radius of 100 m and 500 m around the sampling site. Correlating these measures with specific sites quantified urbanisation more precisely than using a spatial gradient from an urban core to the outskirts^97^. Measurements of impervious surface, human population density and density of roads were taken from the Senate Department for Urban Development and Housing^98^ using a geographic information system (GIS). As all three variables, and both radii, were highly correlated (r > 0.9), we used the amount of impervious surface in a 500 m radius as the urbanisation measure (min = 1.19%, max = 62.78%). To test for potentially confounding effects of vegetation related parameters, we assessed coverage of herbal layer, total and alien plant species richness; the latter parameter included species introduced (neophytes) since 1492. Vegetation data were sampled in vegetation relevés (4 × 4 m), following the standard approach of Braun-Blanquet^99^.

We applied a standard method for investigating plant-pollinator interactions by exposing vases with flowering branches to potential pollinators^18^. To reduce wilting, flowering branches of both species were taken from plants from one donor site close to the study sites and immediately placed into water-filled flower vases. Four vases with equal numbers of flowering branches of *Robinia* and *Cytisus* (8 vases in total) were alternately placed 90 cm apart from one another in a square, with observers located nearby. After counting pollinators, blossoms per vase were counted to ensure standardisation for statistical analyses.

### Data collection

In June 2017, pollinator counts were carried out under good weather conditions^100^, with clear skies, wind speed at 1–1.4 m/s and warm temperatures (≥22 °C). We measured air temperature, air humidity and wind speed using a hand anemometer (M0198652 Handheld USB Thermo-Hygro-Anemometer). The observations started around noon. While observation periods of 15 minutes have led to reasonable results in a comparable study^44^, we extended the time to 45 minutes to enhance accuracy of data. We were only able to sample each site once because optimal sampling conditions were limited to 4 days, due to a period of heavy rainfall, and because of rapid wilting of *Robinia* flowers.

Flower visits were differentiated into 3 categories (Fig. 1): immediate flower access, hovering around flowers with subsequent flower access, and hovering without flower access. Summation of these categories provided the total number of flower visits. Based on flower access after hovering, we also determined a decision rate for visits to a particular species by dividing the number of times flowers were accessed after hovering by the hovering count. During pollinator counting we distinguished optically between the following pollinator taxa: honey bees (*Apis mellifera*), bumblebees (*Bombus* spp.), wild bees (Apidae, but not members of the genera *Apis* and *Bombus*), wasps (Apocrita), hoverflies (Syrphidae), other flies, mosquitoes (Diptera), beetles (Coleoptera), and butterflies (Lepidoptera). When honey bees and wild bees could not be distinguished correctly both taxa were merged as Hymenoptera s. l. This is a method for assessing pollinator visits to plants that has previously been used^101^.

### Statistical analysis

Prior to analyses, we standardised pollinator counts and calculated contacts or hovering, respectively, per 100 blossoms. To determine the relative attractiveness of *Robinia* or *Cytisus* we compared the number of direct contacts, the number of hovering instances and the decision rate using GLMM (R function lmer) taking a Gaussian distribution. Data were log-transformed before and distribution was checked graphically using diagnostic plots^102^. Location was considered a random effect to meet the requirements for testing paired samples. To test, if attractiveness of the alien vs. native plant for pollinators changed along an urbanisation gradient, we first calculated the ratio for direct contacts, hovering and decision ratio between *Robinia* and *Cytisus*. Smaller ratios indicated a higher attractiveness for *Robinia* blossoms, and vice versa. We analysed effects of urbanisation (% impervious area in a 500 m radius) and vegetation variables (number of plant species and of neophytes) on immediate blossom access, only hovering around flowers, and decision ratios using a GLM with Gaussian distribution. Backward selection of variables was based on Akaike information criterion (AIC) values, where the lowest AIC denoted the best model.

## Supplementary information

Appendix 1: Spatial and environmental data at study sites.Appendix 2: Total counts for pollinator taxa interacting with flowering branches of the invasive alien plant Robinia pseudoacacia and the native plant Cytisus scoparius. Diptera s. l. comprised all flies except Syrphidae. Insects were assigned to Hymenoptera s. l. when differentiation between honey bees and wild bees was not possible.

## Data Availability

The supplementary data is included as an appendix.
