# Supplementary material for: Urbanisation modulates plant-pollinator interactions in invasive vs. native plant species
Source: Sci Rep. 2019 Apr 23;9:6375. doi: 10.1038/s41598-019-42884-6 (PMC6477046; doi:10.1038/s41598-019-42884-6)
Supplement: Supplementary file 1 — Appendix 1: Spatial and environmental data at study sites.Appendix 2: Total counts for pollinator taxa interacting with flowering branches of the invasive alien plant Robinia pseudoacacia and the native plant Cytisus scoparius. Diptera s. l. comprised all flies except Syrphidae. Insects were assigned to Hymenoptera s. l. when differentiation between honey bees and wild bees was not possible. [file 41598_2019_42884_MOESM1_ESM.pdf]

Urbanisation modulates plant-pollinator interactions in invasive vs. native plant species

Sascha Buchholz & Ingo Kowarik

Appendix 1: Spatial and environmental data at study sites.

ID	Location	Percentage		Number of		Percentage
	Long / lat	Vegetation	Herbal layer	Plant species	Neophytes	Impervious area
1	13.30888/52.40597	99	70	33	2	15.03
2	13.24265/52.48820	95	45	26	2	4.38
3	13.37276/52.48854	75	40	29	4	62.49
4	13.38538/52.53438	75	55	32	7	62.78
5	13.37698/52.53825	100	70	29	1	61.56
6	13.35908/52.46165	99	50	25	2	32.73
7	13.19511/52.47137	95	75	31	3	6.11
8	13.09988/52.41562	96	70	26	3	6.69
9	13.24726/52.58265	85	60	31	2	1.19
10	13.31621/52.45679	100	90	NA	NA	59.07

Appendix 2: Total counts for pollinator taxa interacting with flowering branches of the invasive alien plant *Robinia pseudoacacia* and the native plant *Cytisus scoparius*. Diptera s. l. comprised all flies except Syrphidae. Insects were assigned to Hymenoptera s. l. when differentiation between honey bees and wild bees was not possible.

**Taxon**	***Robinia pseudocacia***			***Cytisus scoparius***		
	Total Contacts	Immediate access	Hovering	Total Contacts	Immediate access	Hovering
Coleoptera	3	1	2	9	7	2
Lepidoptera	2	0	2	0	0	0
Hymenoptera s. l.	52	39	12	63	44	19
- Apiformes	3	0	3	4	4	0
- Apocrita	1	1	0	1	1	0
- *Bombus* spec.	5	0	5	19	13	6
- *Apis mellifera*	7	2	5	10	5	5
Diptera s. l.	70	33	37	77	58	19
- Syrphidae	4	2	2	4	3	1
